# Establishing a human bone marrow single cell reference atlas to study ageing and diseases

**DOI:** 10.3389/fimmu.2023.1127879

**Published:** 2023-03-15

**Authors:** Nicole Yee Shin Lee, Mengwei Li, Kok Siong Ang, Jinmiao Chen

**Affiliations:** ^1^ Singapore Immunology Network (SIgN), Agency for Science, Technology and Research (A*STAR), Singapore, Singapore; ^2^ Immunology Translational Research Program, Department of Microbiology and Immunology, Yong Loo Lin School of Medicine, National University of Singapore (NUS), Singapore, Singapore

**Keywords:** bone marrow, single-cell atlas, ageing, inflammageing, data integration

## Abstract

**Introduction:**

Ageing in the human bone marrow is associated with immune function decline that results in the elderly being vulnerable to illnesses. A comprehensive healthy bone marrow consensus atlas can serve as a reference to study the immunological changes associated with ageing, and to identify and study abnormal cell states.

**Methods:**

We collected publicly available single cell transcriptomic data of 145 healthy samples encompassing a wide spectrum of ages ranging from 2 to 84 years old to construct our human bone marrow atlas. The final atlas has 673,750 cells and 54 annotated cell types.

**Results:**

We first characterised the changes in cell population sizes with respect to age and the corresponding changes in gene expression and pathways. Overall, we found significant age-associated changes in the lymphoid lineage cells. The naïve CD8^+^ T cell population showed significant shrinkage with ageing while the effector/memory CD4^+^ T cells increased in proportion. We also found an age-correlated decline in the common lymphoid progenitor population, in line with the commonly observed myeloid skew in haematopoiesis among the elderly. We then employed our cell type-specific ageing gene signatures to develop a machine learning model that predicts the biological age of bone marrow samples, which we then applied to healthy individuals and those with blood diseases. Finally, we demonstrated how to identify abnormal cell states by mapping disease samples onto the atlas. We accurately identified abnormal plasma cells and erythroblasts in multiple myeloma samples, and abnormal cells in acute myeloid leukaemia samples.

**Discussion:**

The bone marrow is the site of haematopoiesis, a highly important bodily process. We believe that our healthy bone marrow atlas is a valuable reference for studying bone marrow processes and bone marrow-related diseases. It can be mined for novel discoveries, as well as serve as a reference scaffold for mapping samples to identify and investigate abnormal cells.

## Introduction

The human immune system plays a crucial role in fending off challenges from viruses and microbes, as well as malignancies. As an individual ages, the immune system ages alongside, characterised by immune cell population size changes, functional capability alterations, and mutation accumulation (1). These degradations of the immune system in turn increase the risks of infections and cancers (2, 3). Chronic, low-grade inflammation, or inflammageing, also develops with age and is a risk factor for various diseases including diabetes mellitus and cardiovascular diseases (4, 5). As the bone marrow (BM) is the main source of new immune cells, it is important to study ageing-related changes in the bone marrow and how they contribute to the weakening of the immune system.

To date, most immune ageing studies employed flow cytometry to analyse peripheral blood samples (1). Flow cytometry has high throughput and is low cost compared to sequencing experiments but can only measure 20+ parameters, or 40+ parameters for destructive mass cytometry. To probe transcriptome-wide changes, sorted cells can be subjected to bulk sequencing but this limits the analysis of differences to predefined cell types. Mouse samples are also commonly used as they are the easiest to obtain. For human-based studies, the reported age-associated changes in peripheral blood cell population abundance include shrinking naïve T cell populations, increasing effector/memory and regulatory T cell populations, shrinking B cell subsets, and increasing monocyte populations (1). Overall, there is also a clonal shift towards myeloid-biased hematopoietic stem cells (HSCs), which result in a skew towards circulating myeloid populations (6).

In the past decade, advances in single-cell technologies have lowered costs while increasing the scale of data generated. This has spurred an increasing number of studies exploiting single-cell RNA sequencing (scRNA-Seq) to profile different tissues including the bone marrow. The consequent explosion of publicly available data affords us opportunities to construct large scale cell atlases from a wide array of sample datasets. To date, several human bone marrow atlases have been constructed. Most incorporate both healthy and diseased samples to investigate the differences between them with only a handful of studies that focus on healthy bone marrow. Hay et al. sequenced over 100,000 cells from eight healthy donors, spanning 35 annotated cell types (7). They also characterised the immunological differences due to gender and age. They detected minimal gender-specific differences but HSC frequency was found to reduce with age. However, the low number of healthy donors makes it difficult to establish strong and generalisable conclusions. The Human Cell Atlas also hosts a larger updated census of more than half a million immune cells from samples that include bone marrow and umbilical cord blood (8). However, there currently appears to be no associated analysis published.

Here we present our healthy bone marrow atlas constructed with 145 publicly available scRNA-Seq datasets from 22 studies. As a single cell resource, it can be mined to gain insights into healthy bone marrow tissue and serve as a reference onto which we can map disease samples to investigate disease pathology. We first employed it to investigate ageing-related changes in the bone marrow. We identified cell populations that proportionally change with age and the related changes in gene expression and associated pathways. We then trained an age predictor model with cell type-specific ageing gene signatures to investigate the apparent age of disease samples with respect to their chronological age. There we found acute myeloid leukaemia (AML) samples to have a lower apparent age for more aged samples while younger samples had a higher predicted age. Finally, we mapped blood cancer samples onto the atlas to identify abnormal cell types and associated transcriptomic changes. We were able to identify abnormal plasma cells and erythroblasts in multiple myeloma (MM) samples, and abnormal cells in acute myeloid leukaemia samples.

## Results

### Construction of human healthy bone marrow cell atlas and its application in identifying cells and gene signatures associated with ageing and diseases

**Figure 1:**
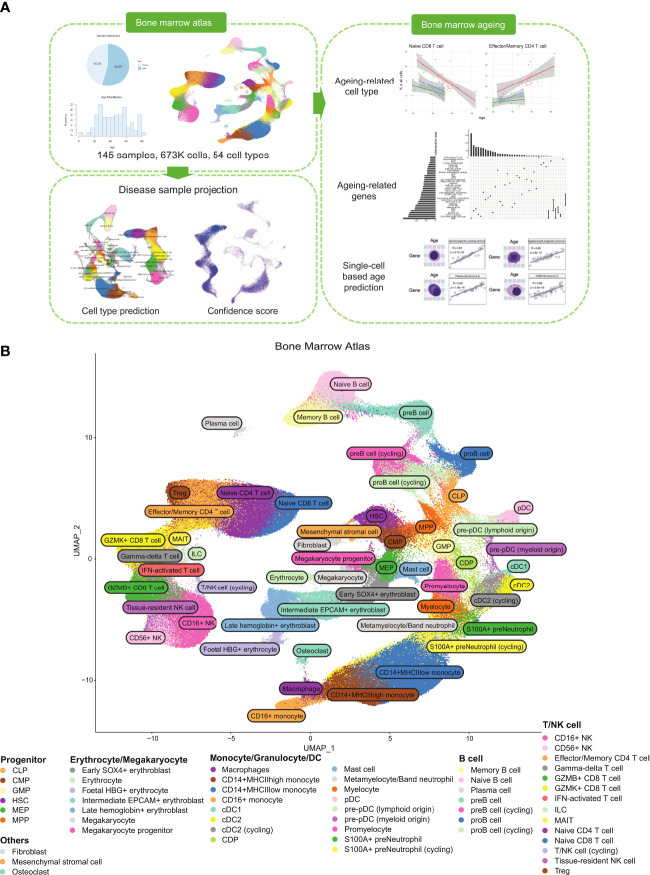
Healthy BM atlas construction, annotation, and analysis. **(A)** Integration and analysis of publicly available healthy BM samples to construct the atlas. 145 healthy BM samples were used to build the final atlas with 673,750 cells in 54 cell types. Blood disease samples of acute myeloid leukaemia and multiple myeloma were projected onto the healthy BM atlas to identify disease-specific populations. Age-correlated cell type frequency changes and associated dysregulated genes and pathways were identified. Identified age-associated gene signatures were then used with machine learning to predict cell age. **(B)** UMAP of the integrated healthy BM atlas with cells coloured by their cell type annotation. For an interactive version of this atlas, please visit DISCO at http://www.immunesinglecell.org/atlas/bone_marrow.

To construct our reference healthy human BM atlas, we used 145 publicly available human BM scRNA-Seq datasets with publicly available sequencing reads from 22 projects (**Figure 1A**). Of the 145 samples, 92 samples have age information, with age ranging from 2 to 84 years and a median of 45 years (**Supplementary Table 1**). Our collected data also includes 34 foetal BM samples. From samples with gender information, the gender ratio is relatively balanced at 54 female and 45 male samples. 91 samples were sorted using a variety of strategies. All samples were sequenced using the 10x Genomics sequencing platforms.

The raw reads were previously processed as part of the DISCO database (9). We employed our pipeline to remap the reads to a single reference genome, GRCh38 (Ensembl 93), for annotation consistency and to reduce potential technical effects. We then processed the resulting read counts using the Seurat package (10). Standard quality control steps on UMI counts, number of detected genes, and the fraction of mitochondrial and ribosomal genes were applied to filter out low quality cells. To remove the significant batch effects present, we employed our FastIntegration tool developed for atlas-scale integration (11) (**Supplementary Figure 1**). This was followed by unsupervised clustering and differential gene expression analysis. We then annotated each cluster’s cell type using canonical marker genes (**Supplementary Figure 2** and **Supplementary Table 2**).

Our constructed healthy BM atlas is currently the largest among existing healthy BM atlases (7, 12, 13) in both cell count and number of donor samples. It consists of 673,750 cells with 54 annotated cell types (**Figure 1B**). The cell types present mostly overlap with the previously constructed healthy BM atlas by Hay et al. (7) but are annotated at higher resolution of cell subtypes with known markers. Our annotated cell types can be divided into five major groups: T/NK cells, B cells, monocytes and DCs, progenitor cells, and erythrocytes/megakaryocytes. Other smaller clusters of cells identified are osteoclasts, fibroblast, and mesenchymal stromal cells. For the T/NK cells, we could divide the naïve population into CD4^+^ and CD8^+^ subtypes, the CD8^+^ T cells into GZMK and GZMB subtypes, and NK cells into CD16^+^ and CD56^+^ subtypes. We also identified mucosal-associated invariant T (MAIT), gamma-delta T, and regulatory T (Treg) cells. For B cells, we could identify the subtypes along its developmental path from the common lymphoid progenitors (CLP) to pro-B, pre-B, naïve B, memory B, and plasma cells. The monocyte populations found were the CD14^+^ and CD16^+^ subtypes, while the dendritic cells were divided into classical dendritic cells (cDCs) and plasmacytoid dendritic cells (pDCs). We compared our annotations with available annotations of the contributing samples (GSE185381 (14) and Census of Immune Cells (8)) and we found good concordance among the major cell types (**Supplementary Figure 3**).

**Figure 2:**
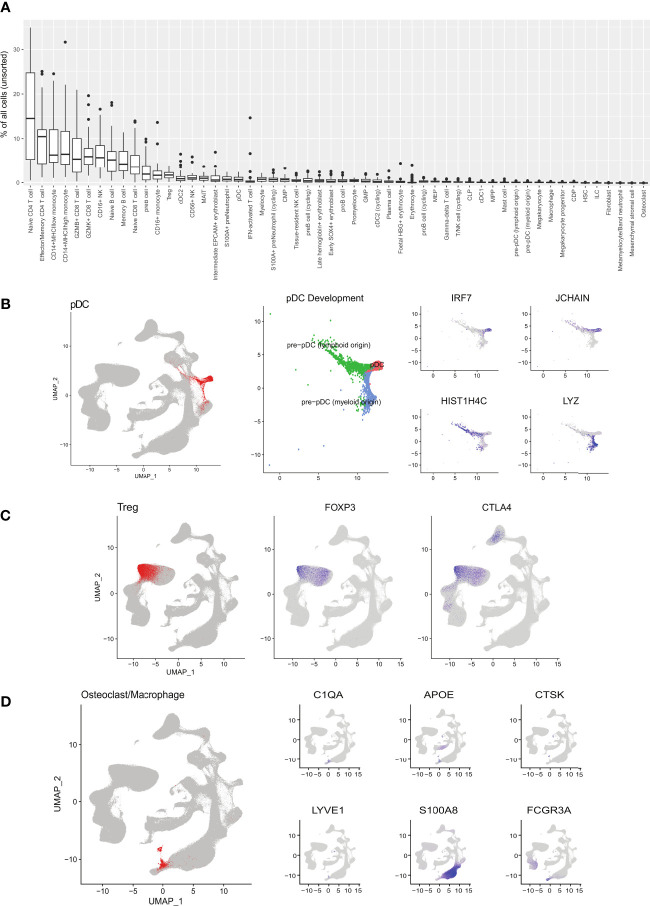
Detailed cell type populations and annotation. **(A)** Percentage of cells for each cell type in unsorted bone marrow samples. **(B)** pDC development pathways of myeloid and lymphoid origins. **(C)** Treg cell population identified by canonical markers. **(D)** Osteoclast/macrophage cells identified by canonical markers.

Using the unsorted samples, we computed the distributions of cell type proportions (**Figure 2A**). Mature T cells formed the majority of cells in the bone marrow with naïve CD4^+^ T cells being the most numerous. Naive CD8^+^ T cells expectedly showed a much lower average percentage with this skew being attributed to the high failure rate of CD8^+^ T cells during selection in the thymus (15). Other cell types, namely monocytes, B cells, and NK cells, made up significant fractions. Overall, these proportions are in line with previous studies on the cell types present in the bone marrow (13).

By integrating a large number of cells into a single atlas, there are sufficient cells to populate the secondary developmental pathways. Here our atlas illustrates the different developmental pathways of pDCs (marked by *IRF7*), namely myeloid and lymphoid origins (16) (**Figure 2B**). The myeloid origin pathway (blue) traces from the DC precursor populations and is marked by the myeloid-associated *LYZ*. The lymphoid origin pathway (green) traced from the CLP population and is marked by *JCHAIN* and *HIST1H4C*. Within the T cell population, we could also distinguish the Treg cell subset (**Figure 2C**). The large cell count enabled us to increase the resolution of clustering and identify rare cell types in the bone marrow, such as osteoclasts (**Figure 2D**) and mesenchymal stromal cells. These cells were not annotated in previous healthy bone marrow atlases. As the integration included foetal data samples, we could identify foetal erythrocytes expressing *HBG1* and *HBG2* (foetal HBG^+^ erythrocyte).

### Bone marrow atlas captures age-related changes in immune cell populations

In ageing studies, blood cell population changes are typically analysed using peripheral blood samples. We first examined cell type frequency changes in the bone marrow and compared them against those reported in human blood samples. Due to large variances in cell populations across samples and studies, we only used samples from three studies, namely GSE120221 with 25 samples (13), GSE185381 with 10 samples (14), and the Census of Immune Cells dataset with 8 samples (8). We selected these studies as they had large numbers of unsorted samples with age information. The remaining studies were either composed of sorted samples or had too few donor samples (<5).

**Figure 3:**
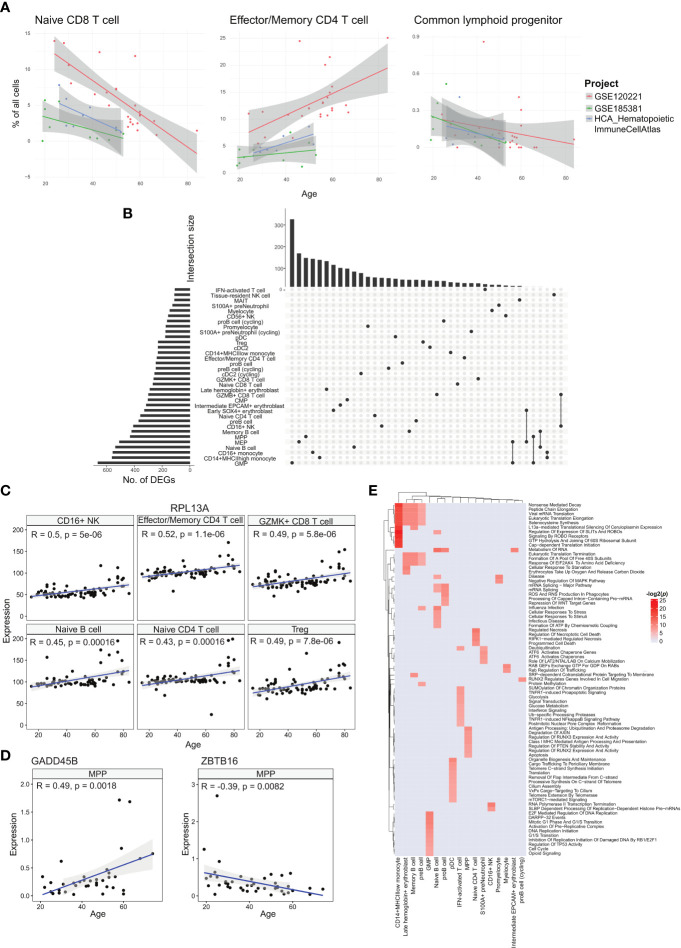
Analyses of age-correlated changes in the bone marrow. **(A)** Regression plots showing significant changes in cell type frequencies with respect to age for naïve CD8^+^ T cell, effector/memory CD4^+^ T cell, and common lymphoid progenitor in the bone marrow. **(B)** UpSet plot with set size depicting the number of significant age-related DEGs for each of the respective cell types and intersection size illustrating the number of age-related DEGs unique to a cell type or shared with other cell types depending on which cells have been filled in the respective columns. **(C)** Regression plots showing the upregulation of sample averaged *RPL13A* expression for multiple cell types across age. **(D)** Regression plots of sample averaged expression of *GADD45* and *ZBTB16*. **(E)** Heatmap of age-associated dysregulated pathways in different cell types.

We tested the correlations for each annotated cell type using a linear model with the study batch as covariate. Two cell types, effector/memory CD4^+^ T and CD16^+^ NK cells, had statistically significant positive correlations across all samples (**Figure 3A**, **Supplementary Table 3**). The effector/memory CD4^+^ T cell’s correlation was statistically significant when combining the regression output from all three studies and even within the GSE120221 and Census study sets, giving us the greatest confidence in this result. Moreover, this correlation has also been found in peripheral blood by Li et al. (17). Increasing proportions of CD16^+^ NK cells have also been reported in the blood of elderly individuals (18).

Among the annotated cell types negatively correlated with age, the naïve CD8^+^ T cell population had the largest average coefficient and was consistent across the three studies. Decline in naïve T cell populations have been reported for peripheral blood in different studies (17, 19, 20) and is well recognised. For the corresponding naïve CD4^+^ T cell population, we obtained a negative correlation with the GSE120221 and GSE185381 sets, but a positive correlation with the Census study set (**Supplementary Figure 4**). The latter result is unexpected and can be explained by the small number of samples (n=8) coupled with high variability within the data that can mask the expected age-related decline. Another notable cell type negatively correlated with age is the CLP population. As progenitor cell types, changes in these populations have disproportionate impact on mature cell type populations and consequently the immune system’s functions. This decline in the proportion of lymphoid-biased progenitors and consequent myeloid bias in haematopoiesis is widely reported (21). The computed correlations for all cell types are given in **Supplementary Table 3**.

### Cell type-specific ageing gene signature and pathway changes correlated with ageing

We next investigated the cell type-specific genes with expression that correlate with age, using all samples with age metadata (**Figure 3B**). Among the annotated cell types, GMP cells had the largest number of age-associated differentially expressed genes (DEGs) with 663 genes, while IFN-activated T cells had the least with 107 genes. Most of these genes were shared among few cell types, with only 20 genes shared in 10 or more cell types. The most conserved DEG was *RPL13A* in 17 cell types (**Figure 3C**). *RPL13A*’s upregulation with age has also been reported for almost all tissue types of mice (22). Other genes of note include *IMMP2L* and *DIP2B* which showed downregulation with age in 10 cell types. Switching off *IMMP2L* signalling has been shown to drive cell senescence (23). Similarly, *DIP2B* knockout cells expressed senescence markers found in ageing cells (24). We also found positive age correlations for chemokines *CCL4*, *CCL4L2*, and *CCL5* in tissue-resident NK cells, *CCL4L2* in CD16^+^ NK cells, *CCL4* in CD56^+^ NK cells, and *CCL3* in GZMK^+^ CD8^+^ T cells (**Supplementary Table 4**).

With ageing, the skew towards myeloid lineage haematopoiesis is well documented (25). Within the MPP population, we investigated transcriptomic changes that can contribute to the decline in differentiation towards the lymphoid lineage. Here we found two notable genes, *GADD45B* which was upregulated with respect to age, and *ZBTB16* that was downregulated (**Figure 3D**). *GADD45B* has been characterised as a myeloid differentiation gene and plays a role in the response of myeloid cells to stress stimulation (26, 27), while the *ZBTB16* gene is a negative regulator of myeloid cell development (28).

We followed up with pathway analysis of the age correlated genes using EnrichR (29). Here we show the enriched Reactome pathways (**Figure 3E**). The different cell types show a wide range of dysregulated pathways. We note that the CD14^+^ MHCII^low^ monocytes, late haemoglobin^+^ erythrocytes, memory B cells, and pre-B cells similarly show dysregulated translation processes. Other B cell subtypes, namely pro-B cells and naïve B cells, show dysregulated mRNA processing pathways, and the naïve B cell subset also show dysregulated cellular stress pathways. For the interferon-activated T cells, the dysregulated pathways are primarily centred around metabolism and interferon signalling. The metabolic changes in T cells due to ageing is linked to cell senescence and reduced functionality, though the exact mechanisms are under investigation (30).

### Age prediction of disease samples show divergence from chronological age

**Figure 4:**
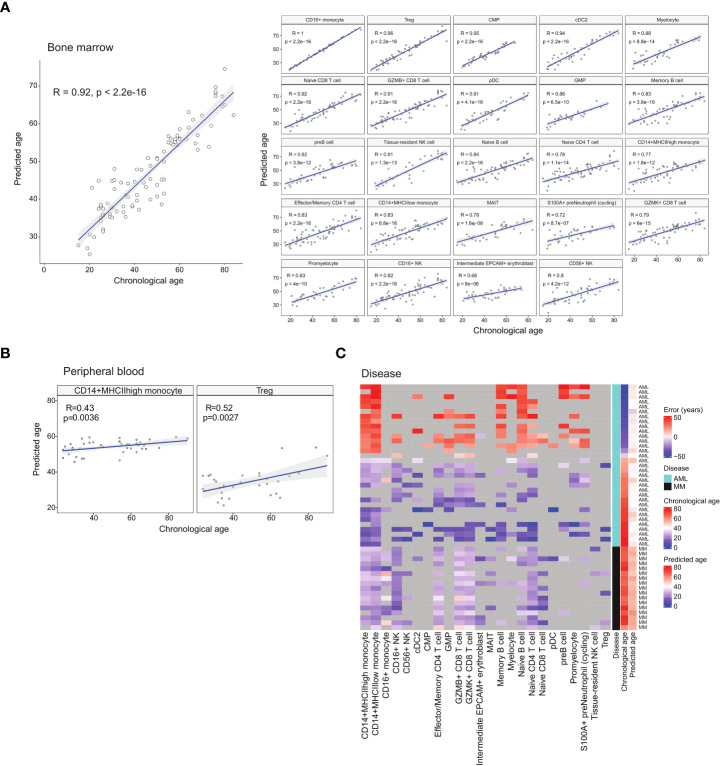
Age prediction using identified age-associated DEGs with machine learning. **(A)** Regression plots showing the correlation between chronological age and median predicted age of the respective cell types in healthy BM samples. **(B)** Regression plots showing the correlation between chronological age and median predicted age of select peripheral blood cell types. **(C)** Heatmap depicting the differences (error) between chronological age and predicted age of diseased BM samples.

Cancer is generally recognised as an ageing-related disease. While ageing increases the risk of cancer, it has also been reported that cancer can alter the expression trends of ageing-related genes (31). We theorised that diseases including cancer can modify the gene expression of diseased cells to appear younger or older than their chronological age. Thus, we investigated how diseased cells’ age predicted by their transcriptome differ from their chronological age. We first trained a cell age predictor using the healthy atlas. The predictor was constructed using elastic net regression and the age-related DEGs identified for each cell type. For each sample, we computed the age of each cell type present, and the median predicted age was used as the overall predicted age. The feature gene set were selected *via* regularisation in the elastic net regression, and we employed 10-fold cross validation to check the hyperparameters (**Supplementary Figure 5**). The resulting predictor was able to predict the ages of healthy individuals with a correlation coefficient of 0.92 and an error of 6.3 years (**Figure 4A**). We further tested our predictor using the corresponding cell types from healthy blood samples, obtaining good predictions with CD14^+^ monocytes and Treg cells (**Figure 4B**). List of blood samples are presented in **Supplementary Table 5**.

We then used the age predictor to predict the age of patients diagnosed with acute myeloid leukaemia (AML) and multiple myeloma (MM). The predicted ages of the cancer samples showed the trends of older patients being predicted to have a younger age while younger patients were predicted to have an older age (**Figure 4C** and **Supplementary Table 6**). For the AML samples, the predicted ages fall in the range of 32 to 51 years old with an average of 41.8. For the MM samples, the predicted ages fall in the range of 49 to 60 years old with an average of 53. This suggests that cancer alters gene expression patterns to portray a cancer-specific apparent age that may be different across different cancer types. In the case of AML, this also affects the paediatric disease subtypes. Future investigation into this apparent cell age phenomenon will encompass other blood cancers.

### Blood cancer-specific cells identified by mapping disease samples onto atlas

**Figure 5:**
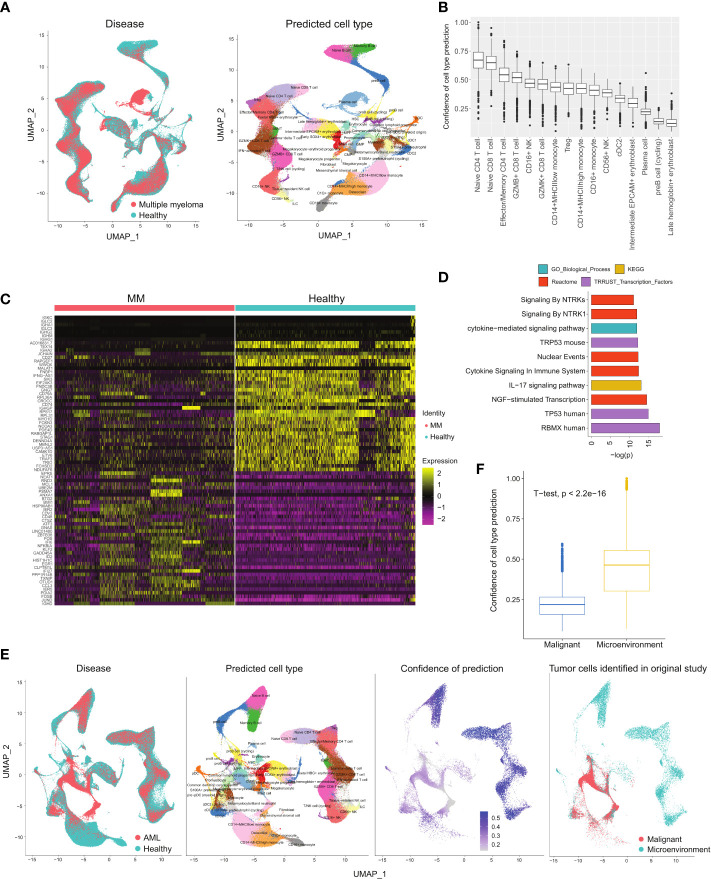
Projecting diseased BM samples onto healthy reference to identify disease-specific cells. **(A)** Multiple myeloma datasets projected onto the healthy BM atlas. **(B)** Confidence scores of cell type prediction of MM samples. **(C)** Heatmap of DEGs between plasma cells from MM samples versus healthy samples. **(D)** Pathway enrichment analysis of MM plasma cell DEGs. **(E)** Projection of AML datasets onto the healthy BM atlas. **(F)** Confidence scores of cell type prediction with AML samples.

To demonstrate our healthy BM atlas’ applicability as a reference for identifying diseased cells, we mapped samples of AML and MM onto our atlas. By integrating healthy and diseased samples together, abnormal cells can be identified. We first mapped 10 MM samples from GSE189460 onto our healthy atlas and performed label transfer to annotate the cells (**Figure 5A**). We also computed a cell type prediction confidence score based on each cell’s distance to its neighbours in the reference atlas (Methods). Lower prediction confidence scores denote mapped cells that were phenotypically different from cells found in the healthy atlas, which in this case implied diseased cells. Among the mapped cells, the lowest scores were found among the predicted plasma and erythroblast cells (**Figure 5B**). The predicted plasma cell types correspond to the malignant plasma cells that accumulate in the bone marrow while abnormalities in the erythroblast compartment correspond to the disrupted erythropoiesis process which gives rise to the common MM symptom of anaemia (32).

We compared the malignant plasma cells to their healthy counterparts and obtained 80 DEGs (p< 0.05 and |logFC| > 0.5) (**Figure 5C** and **Supplementary Table 7**). The upregulated genes include the previously reported *JUND* (33), *FOSB*, *PDIA2* (34), and *CCL3* (35). Elevated levels of *CCL3* in the bone marrow is of particular interest as it has been proposed to suppress erythropoiesis and cause anaemia in MM patients (36). In the list of downregulated DEGs, low *CD27* expression in malignant plasma cells has been reported to be correlated with poor prognosis (37) while the tumour suppressor gene *WWOX* has been found downregulated due to translocations or deletions (38). In the enrichment analysis, dysregulated pathways include *TP53* and cytokine signalling (**Figure 5D**). The dysregulation of cytokine signalling is an expected characteristic of malignant plasma cells due to the high levels of cytokines that also serve as chemoattractants that attracts them to the bone marrow (39).

We next mapped 10 AML samples from GSE185381 (14) onto our BM atlas (**Figure 5E**). We performed label transfer to label the AML sample cells and computed the prediction confidence score for each cell. Comparison of the computed scores with the malignant cell labels in the original annotation showed good correlation with the malignant cells have low scores (**Figure 5F**). The malignant cells’ transferred labels are primarily progenitor types and myeloid types (monocytes and dendritic cells), which have been previously reported (40). This presents an approach towards identifying malignant cells as an alternative to employing mutation detection.

## Discussion

The bone marrow is the site of haematopoiesis, a highly regulated process that must be responsive to the body's needs. Like other bodily processes, haematopoiesis is profoundly affected by ageing. Well described by multiple studies, the ageing process brings about many changes to the cell populations in the bone marrow, including shrinking naïve cell populations, accumulating memory cell populations, and myeloid bias in cellular output (1). In this work, we constructed a reference healthy BM atlas using published scRNA-Seq datasets with publicly available reads. The samples’ ages spanned the lifespan of most individuals, from 2 to 84 years of age, with 33 additional foetal samples. We first investigated age-related changes in the BM. We recapitulated some of the reported cell population changes with respect to age and identified accompanying gene expression changes. Among the age-correlated genes, most were cell type-specific with only a small number being shared among the different cell types. Notably, we found the downregulation of *IMMP2L* and *DIP2B*, which have been implicated in cell senescence. We also found chemokines upregulated in different NK cell subtypes. Future work will aim to refine the ageing-associated genes and pathways, and investigate the mechanisms linking to reduced immune functions.

In the BM atlas constructed by Human Cell Atlas consortium (7), HSC cell frequency decreased with age. This was not replicated in our analysis. This discrepancy may be explained by HSCs representing a very small percentage of each sample (<4%), making the measured frequency prone to errors. Thus, a much larger number of samples and larger cell counts per sample are needed to reduce the error margins and verify this observation. Moreover, the HCA atlas used only eight samples from donors between the ages of 25 and 53, which limited the study’s statistical power.

We also employed the cell type-specific genes that were correlated with age to train a cell age predictor. With the predictor, we found chronologically younger blood cancer samples to have an older predicted age while older blood cancer samples showed a younger predicted age. As cancer cells possess characteristics of immortality, active cell division, and higher metabolic activity, they can appear phenotypically younger. For chronologically younger samples, the genomic and even environmental changes associated with cancer may also alter gene expression to seemingly reflect an older cellular age. Higher stress levels than healthy tissue, cellular dysregulation, and activated but ineffectual DNA repair pathways can be contributing factors as well.

Most disease studies construct cell atlases that combine both healthy and diseased samples to study the differences between them. However, a comprehensive healthy tissue atlas is still valuable to disease studies. As recently demonstrated by Dann et al. (41), using a separate healthy reference as a reference scaffold to map disease samples and matched healthy controls can improve the identification of disease-associated cell states and reduce the number of control samples while preserving the rate of false discoveries. Here, our constructed healthy BM atlas encompassing a large number of donor samples from multiple studies and across a wide age range, is a comprehensive healthy reference that can serve as a baseline for comparative studies with diseased samples. As bone marrow samples require an invasive procedure to obtain, using our healthy atlas to reduce the needed number of healthy control samples is greatly beneficial. For this work, we demonstrated the mapping of disease samples onto our atlas to identify diseased cell states. We identified abnormal plasma cells and erythroblasts in MM samples, as well as AML-specific abnormal cells. The diseased cells can be easily identified by their low cell type prediction confidence scores. We further inspected the differentially expressed genes of the MM plasma cells and identified previously reported disease markers.

We believe that our BM atlas is a valuable reference for studying healthy bone marrow processes and bone marrow-related diseases. As more BM scRNA-Seq data become available, we will continuously update and improve on our atlas currently available in the DISCO (11) atlas collection: http://www.immunesinglecell.org/atlas/bone_marrow. We will also pursue future studies on bone marrow diseases using our atlas as the reference scaffold.

## Methods

### Single-cell RNA-Seq data collection, integration, and annotation

We retrieved healthy bone marrow sample datasets from the DISCO database (9). The datasets were previously preprocessed from raw reads and mapped onto the human reference genome, GRCh38 (Ensembl 93). Except for data integration with FastIntegration, we employed the Seurat package (10) for downstream data analyses. For each sample, we filtered the cells based on their unique molecular identifier (UMI) counts, the number of detected genes, and the fraction of mitochondrial and ribosomal genes. As the data were acquired in different experiments, we utilised the distribution of QC metrics from each sample to manually determine the cut-offs applied for filtering. Subsequently, samples with less than 200 cells were removed and the gene expression for each cell was normalised to the total expression using the “NormalizeData” function found in the Seurat package.

We next applied our FastIntegration algorithm to integrate the retained data samples. For each sample, we first identified the top 3000 highly variable features. Thereafter, we merged the lists from all samples and selected the top 3000 most common ones. These highly variable features were used to identify the anchors between samples and the anchors were then used for the subsequent integration and batch correction steps. After integration, the batch-corrected gene expression values were standardised using the “ScaleData” Seurat function and Principal Component Analysis (PCA) was performed. The first 30 PCs were then used for Uniform Manifold Approximation and Projection (UMAP) to enable visualisation. For clustering, we built the KNN graph based on the Euclidean distance in PCA space and applied the Louvain algorithm. The Wilcoxon rank sum tests were used for identifying differentially expressed genes (DEGs) in each cluster, which were subsequently utilised for manual cell type annotation.

### Identification of age-related cell types

We selected three projects (GSE185381, GSE120221, and HCA_HematopoieticImmuneCellAtlas) that had unsorted data and sample sizes larger than 5. For each cell type, we performed linear regression on the proportion of each cell type with age with the following model:


$$x_{j}=\beta_{0}Age_{j}+\beta_{1}Project_{j}$$

where *x_j_* is the percentage in sample *j*. A Spearman correlation *p* value was computed for each project and an overall *p* value for the linear model.

### Age-related gene identification and enrichment analysis

To identify age-correlated genes, we averaged the batch-corrected gene expression values of each cell type in each sample. We only considered cell types found in at least 20 samples and with at least 20 cells. Only the genes expressed in > 10% cells were used. Subsequently, we correlated gene expression with age using the Pearson correlation and retaining genes with *p* value less than 0.01 as age-related genes. For gene set enrichment analysis, we used Enrichr (29) with gene sets from the KEGG, GO, Reactome, and TRUST databases. Pathways with an adjusted *p* value less than 0.01 were selected for visualisation. We also compared our identified age-related genes with the gene list in GenAge's database (42) and genes identified in epigenetic clocks (43).

### Age prediction

Based on the identified age-related genes, we trained a model to predict the age of a sample. As the age-related genes we identified were linearly correlated with age, we applied elastic net linear regression from the glmnet package (44) to build an age prediction model for each cell type. The performance of this model was evaluated using 10-fold cross validation (**Supplementary Figure 5**), and the median predicted age of all cell types was taken as the predicted age for the sample. To estimate the model’s accuracy, we calculated the median absolute difference between the predicted age and chronological age. Prior to predicting the age of new cell samples, we first mapped them to the reference atlas and using only the cells that were confidently mapped for the age prediction.

### Mapping of diseased samples to bone marrow atlas

We downloaded single-cell RNA-Seq data of acute myeloid leukaemia and multiple myeloma samples from the DISCO database. For each disease, we first integrated the data of all samples using FastIntegration. Subsequently, another round of integration was performed to integrate the diseased samples together with the healthy bone marrow atlas with the latter serving as the reference. Finally, we used the integrated data for PCA, UMAP generation, and clustering. We annotated the disease sample cells based on the most prevalent cell type among its 30 nearest neighbours in the reference atlas. We then compute a confidence score by taking the inverse of the distance between each cell and its 30 nearest neighbours, and then normalising it to a range of 0 to 1. The confidence scores were fit into a two-component Gaussian mixture distribution, with cells in the first component being deemed as confidently assigned.

## Data availability statement

The original contributions presented in the study are included in the article/**Supplementary Materials**. Further inquiries can be directed to the corresponding author. The scripts used in the study can be downloaded from the Github repository: https://github.com/JinmiaoChenLab/Bone_Marrow_Aging.

## Author contributions

JC conceptualised and supervised the study. ML and NL analysed the data, annotated the cell types, developed the age predictor, and generated the figures and tables. KA, NL, JC, and ML wrote the manuscript. All authors contributed to the article and approved the submitted version.

## Supplementary material

Supplementary Figure 1
**(A)** UMAPs of healthy BM samples before and after integration, coloured by project ID. **(B)** Comparison of cell type labels in the original studies (left) and our constructed bone marrow atlas (right).

Supplementary Figure 2 Cell type-specific DEG heatmaps. Heatmap of top DEGs for each labelled cell type.

Supplementary Figure 3 Cell type annotation comparison. Heatmap comparing cell type annotation of constructed atlas and annotation from original samples.

Supplementary Figure 4 Regression plot of naïve CD4^+^ T cell age correlation. Regression plots of cell type frequency changes with respect to age for all cell types in the bone marrow for naïve CD4^+^ T cells.

Supplementary Figure 5 Errors for 10-fold cross validation of cell age predictor. Median error of prediction computed in the 10-fold cross validation of cell age predictor.

Supplementary Table 1 Data sample metadata.

Supplementary Table 2 Cell type-specific differentially expressed genes.

Supplementary Table 3 Cell type correlations with age.

Supplementary Table 4 Cell type-specific age-correlated genes.

Supplementary Table 5 List of peripheral blood samples used for age prediction.

Supplementary Table 6 Age predictions of diseased samples.

Supplementary Table 7 Multiple myeloma malignant plasma cell DEGs.

## References

[1] Mogilenko DA Shchukina I Artyomov MN. Immune ageing at single-cell resolution. Nat Rev Immunol (2022) 22:484–98. doi: 10.1038/s41577-021-00646-4 PMC8609266 34815556

[2] Franceschi C Campisi J. Chronic inflammation (inflammaging) and its potential contribution to age-associated diseases. J Gerontol A Biol Sci Med Sci (2014) 69 Suppl 1:S4–9. doi: 10.1093/gerona/glu057 24833586

[3] Leonardi GC Accardi G Monastero R Nicoletti F Libra M. Ageing: From inflammation to cancer. Immun Ageing (2018) 15:1. doi: 10.1186/s12979-017-0112-5 29387133 PMC5775596

[4] Wang X Bao W Liu J Ouyang Y-Y Wang D Rong S. Inflammatory markers and risk of type 2 diabetes: A systematic review and meta-analysis. Diabetes Care (2013) 36:166–75. doi: 10.2337/dc12-0702 PMC3526249 23264288

[5] Ferrucci L Fabbri E. Inflammageing: Chronic inflammation in ageing, cardiovascular disease, and frailty. Nat Rev Cardiol (2018) 15:505–22. doi: 10.1038/s41569-018-0064-2 PMC6146930 30065258

[6] Matteini F Mulaw MA Florian MC. Aging of the hematopoietic stem cell niche: New tools to answer an old question. Front Immunol (2021) 12:738204. doi: 10.3389/fimmu.2021.738204 34858399 PMC8631970

[7] Hay SB Ferchen K Chetal K Grimes HL Salomonis N. The human cell atlas bone marrow single-cell interactive web portal. Exp Hematol (2018) 68:51–61. doi: 10.1016/j.exphem.2018.09.004 30243574 PMC6296228

[8] Regev A Li B Kowalczyk MS Dionne D Tickle T Lee J. Census Immune Cells (2020).

[9] Li M Zhang X Ang KS Ling J Sethi R Lee NYS. DISCO: A database of deeply integrated human single-cell omics data. Nucleic Acids Res (2022) 50:D596–602. doi: 10.1093/nar/gkab1020 PMC8728243 34791375

[10] Hao Y Hao S Andersen-Nissen E Mauck WM 3rd Zheng S Butler A. Integrated analysis of multimodal single-cell data. Cell (2021) 184:3573–3587.e29. doi: 10.1016/j.cell.2021.04.048 34062119 PMC8238499

[11] Li M Zhang X Ang KS Chen J. FastIntegration: a versatile r package for accessing and integrating large-scale single-cell RNA-seq data. bioRxiv (2022). doi: 10.1101/2022.05.10.491296

[12] Setty M Kiseliovas V Levine J Gayoso A Mazutis L Pe'er D. Characterization of cell fate probabilities in single-cell data with palantir. Nat Biotechnol (2019) 37:451–60. doi: 10.1038/s41587-019-0068-4 PMC7549125 30899105

[13] Oetjen KA Lindblad KE Goswami M Gui G Dagur PK Lai C. Human bone marrow assessment by single-cell RNA sequencing, mass cytometry, and flow cytometry. JCI Insight (2018) 3:e124928. doi: 10.1172/jci.insight.124928 30518681 PMC6328018

[14] Lasry A Nadorp B Fornerod M Nicolet D Wu H Walker CJ. An inflammatory state remodels the immune microenvironment and improves risk stratification in acute myeloid leukemia. Nat Cancer (2022) 4:27–42. doi: 10.1038/s43018-022-00480-0 36581735 PMC9986885

[15] Sinclair C Bains I Yates AJ Seddon B. Asymmetric thymocyte death underlies the CD4:CD8 T-cell ratio in the adaptive immune system. Proc Natl Acad Sci U S A (2013) 110:E2905–14. doi: 10.1073/pnas.1304859110 PMC3732981 23858460

[16] Reizis B. Plasmacytoid dendritic cells: Development, regulation, and function. Immunity (2019) 50:37–50. doi: 10.1016/j.immuni.2018.12.027 30650380 PMC6342491

[17] Li M Yao D Zeng X Kasakovski D Zhang Y Chen S. Age related human T cell subset evolution and senescence. Immun Ageing (2019) 16:24. doi: 10.1186/s12979-019-0165-8 31528179 PMC6739976

[18] Gayoso I Sanchez-Correa B Campos C Alonso C Pera A Casado JG. Immunosenescence of human natural killer cells. J Innate Immun (2011) 3:337–43. doi: 10.1159/000328005 21576928

[19] Mogilenko DA Shpynov O Andhey PS Arthur L Swain A Esaulova E. Comprehensive profiling of an aging immune system reveals clonal GZMK(+) CD8(+) T cells as conserved hallmark of inflammaging. Immunity (2021) 54:99–115.e12. doi: 10.1016/j.immuni.2020.11.005 33271118

[20] Arthur L Esaulova E Mogilenko DA Tsurinov P Burdess S Laha A. Cellular and plasma proteomic determinants of COVID-19 and non-COVID-19 pulmonary diseases relative to healthy aging. Nat Aging (2021) 1:535–49. doi: 10.1038/s43587-021-00067-x 37117829

[21] Kovtonyuk LV Fritsch K Feng X Manz MG Takizawa H. Inflamm-aging of hematopoiesis, hematopoietic stem cells, and the bone marrow microenvironment. Front Immunol (2016) 7:502. doi: 10.3389/fimmu.2016.00502 27895645 PMC5107568

[22] Zhang MJ Pisco AO Darmanis S Zou J. Mouse aging cell atlas analysis reveals global and cell type-specific aging signatures. Elife (2021) 10:e62293. doi: 10.7554/eLife.62293.sa2 33847263 PMC8046488

[23] Yuan L Zhai L Qian L Huang D Ding Y Xiang H. Switching off IMMP2L signaling drives senescence *via* simultaneous metabolic alteration and blockage of cell death. Cell Res (2018) 28:625–43. doi: 10.1038/s41422-018-0043-5 PMC5993829 29808012

[24] Larsson C Akhtar Ali MA Pandzic T Lindroth AM He L Sjöblom T. Loss of DIP2C in RKO cells stimulates changes in DNA methylation and epithelial-mesenchymal transition. BMC Cancer (2017) 17:487. doi: 10.1186/s12885-017-3472-5 28716088 PMC5513093

[25] Mejia-Ramirez E Florian MC. Understanding intrinsic hematopoietic stem cell aging. Haematologica (2020) 105:22–37. doi: 10.3324/haematol.2018.211342 31806687 PMC6939535

[26] Hoffman B Liebermann DA. Role of gadd45 in myeloid cells in response to hematopoietic stress. Blood Cells Mol Dis (2007) 39:344–7. doi: 10.1016/j.bcmd.2007.06.011 PMC2684334 17686638

[27] Gupta SK Gupta M Hoffman B Liebermann DA. Hematopoietic cells from gadd45a-deficient and gadd45b-deficient mice exhibit impaired stress responses to acute stimulation with cytokines, myeloablation and inflammation. Oncogene (2006) 25:5537–46. doi: 10.1038/sj.onc.1209555 16732331

[28] Vincent-Fabert C Platet N Vandevelde A Poplineau M Koubi M Finetti P. PLZF mutation alters mouse hematopoietic stem cell function and cell cycle progression. Blood (2016) 127:1881–5. doi: 10.1182/blood-2015-09-666974 26941402

[29] Kuleshov MV Jones MR Rouillard AD Fernandez NF Duan Q Wang Z. Enrichr: A comprehensive gene set enrichment analysis web server 2016 update. Nucleic Acids Res (2016) 44:W90–7. doi: 10.1093/nar/gkw377 PMC4987924 27141961

[30] Martin DE Torrance BL Haynes L Bartley JM. Targeting aging: Lessons learned from immunometabolism and cellular senescence. Front Immunol (2021) 12:714742. doi: 10.3389/fimmu.2021.714742 34367184 PMC8334863

[31] Chatsirisupachai K Palmer D Ferreira S de Magalhães JP. A human tissue-specific transcriptomic analysis reveals a complex relationship between aging, cancer, and cellular senescence. Aging Cell (2019) 18:e13041. doi: 10.1111/acel.13041 31560156 PMC6826163

[32] Bouchnita A Eymard N Moyo TK Koury MJ Volpert V. Bone marrow infiltration by multiple myeloma causes anemia by reversible disruption of erythropoiesis. Am J Hematol (2016) 91:371–8. doi: 10.1002/ajh.24291 26749142

[33] Liu R Gao Q Foltz SM Fowles JS Yao L Wang JT. Co-Evolution of tumor and immune cells during progression of multiple myeloma. Nat Commun (2021) 12:2559. doi: 10.1038/s41467-021-22804-x 33963182 PMC8105337

[34] Vatolin S Phillips JG Jha BK Govindgari S Hu J Grabowski D. Novel protein disulfide isomerase inhibitor with anticancer activity in multiple myeloma. Cancer Res (2016) 76:3340–50. doi: 10.1158/0008-5472.CAN-15-3099 27197150

[35] Vallet S Pozzi S Patel K Vaghela N Fulciniti MT Veiby P. A novel role for CCL3 (MIP-1α) in myeloma-induced bone disease *via* osteocalcin downregulation and inhibition of osteoblast function. Leukemia (2011) 25:1174–81. doi: 10.1038/leu.2011.43 PMC4142423 21403648

[36] Liu L Yu Z Cheng H Mao X Sui W Deng S. Multiple myeloma hinders erythropoiesis and causes anaemia owing to high levels of CCL3 in the bone marrow microenvironment. Sci Rep (2020) 10:20508. doi: 10.1038/s41598-020-77450-y 33239656 PMC7689499

[37] Guikema JEJ Hovenga S Vellenga E Conradie JJ Abdulahad WH Bekkema R. CD27 is heterogeneously expressed in multiple myeloma: Low CD27 expression in patients with high-risk disease. Br J Haematol (2003) 121:36–43. doi: 10.1046/j.1365-2141.2003.04260.x 12670329

[38] Aldaz CM Ferguson BW Abba MC. WWOX at the crossroads of cancer, metabolic syndrome related traits and CNS pathologies. Biochim Biophys Acta (2014) 1846:188–200. doi: 10.1016/j.bbcan.2014.06.001 24932569 PMC4151823

[39] Aggarwal R Ghobrial IM Roodman GD. Chemokines in multiple myeloma. Exp Hematol (2006) 34:1289–95. doi: 10.1016/j.exphem.2006.06.017 PMC3134145 16982321

[40] van Galen P Hovestadt V Wadsworth Ii MH Hughes TK Griffin GK Battaglia S. Single-cell RNA-seq reveals AML hierarchies relevant to disease progression and immunity. Cell (2019) 176:1265–1281.e24. doi: 10.1016/j.cell.2019.01.031 30827681 PMC6515904

[41] Dann E Teichmann SA Marioni JC. Precise identification of cell states altered in disease with healthy single-cell references. bioRxiv (2022) 2022:11.10.515939. doi: 10.1101/2022.11.10.515939 PMC10632138 37828140

[42] Tacutu R Thornton D Johnson E Budovsky A Barardo D Craig T. Human ageing genomic resources: New and updated databases. Nucleic Acids Res (2018) 46:D1083–90. doi: 10.1093/nar/gkx1042 PMC5753192 29121237

[43] Horvath S. DNA Methylation age of human tissues and cell types. Genome Biol (2013) 14:R115. doi: 10.1186/gb-2013-14-10-r115 24138928 PMC4015143

[44] Friedman J Hastie T Tibshirani R. Regularization paths for generalized linear models *via* coordinate descent. J Stat Software (2010) 33:1–22. doi: 10.18637/jss.v033.i01 PMC2929880 20808728

