# Supplementary figures and images for: Establishing a human bone marrow single cell reference atlas to study ageing and diseases
Source: Front Immunol. 2023 Mar 15;14:1127879. doi: 10.3389/fimmu.2023.1127879 (PMC10050687; doi:10.3389/fimmu.2023.1127879)

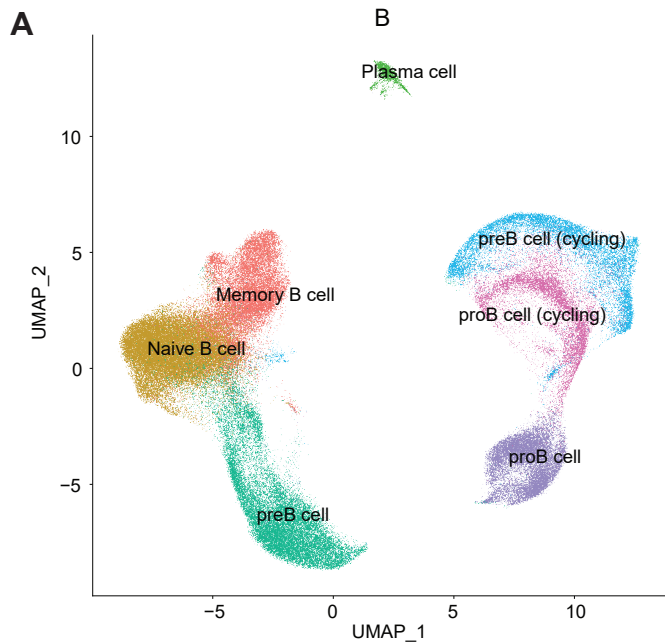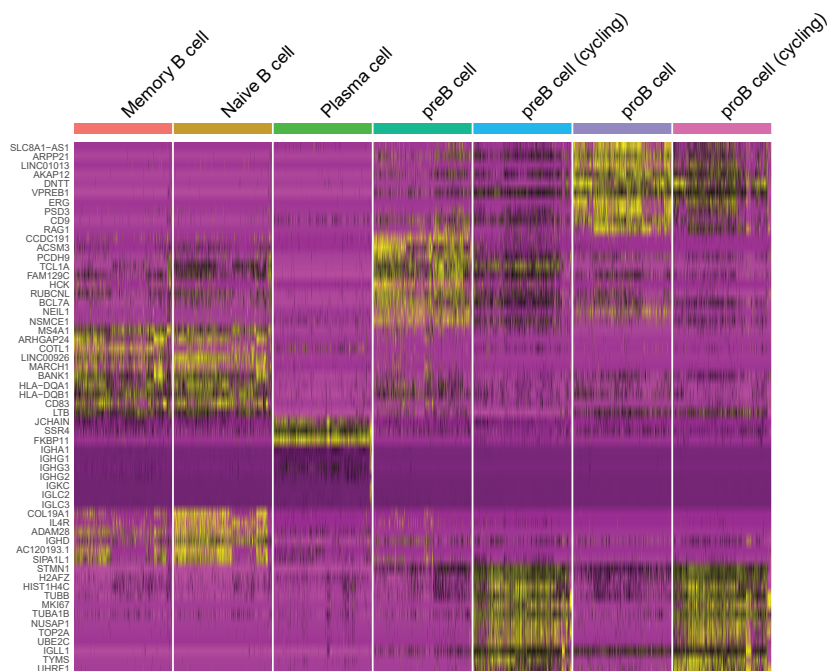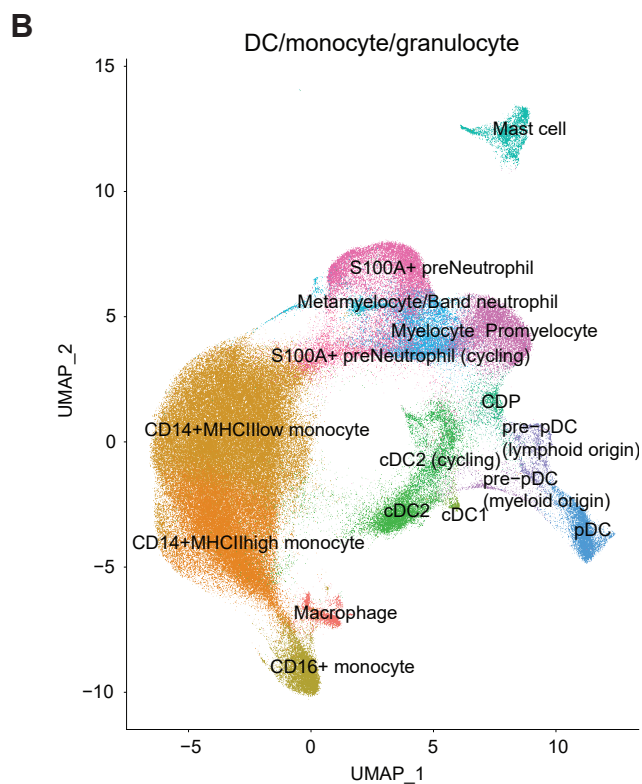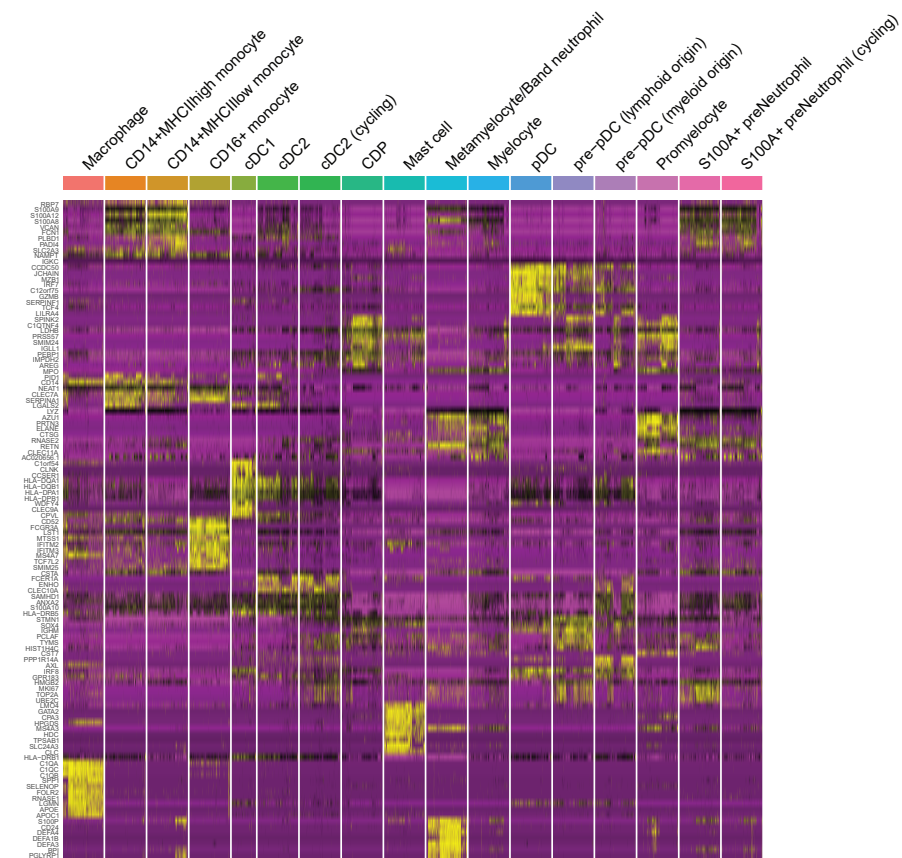

Supplement: Supplementary Figure 2 — Cell type-specific DEG heatmaps. Heatmap of top DEGs for each labelled cell type. [file DataSheet_2.pdf]

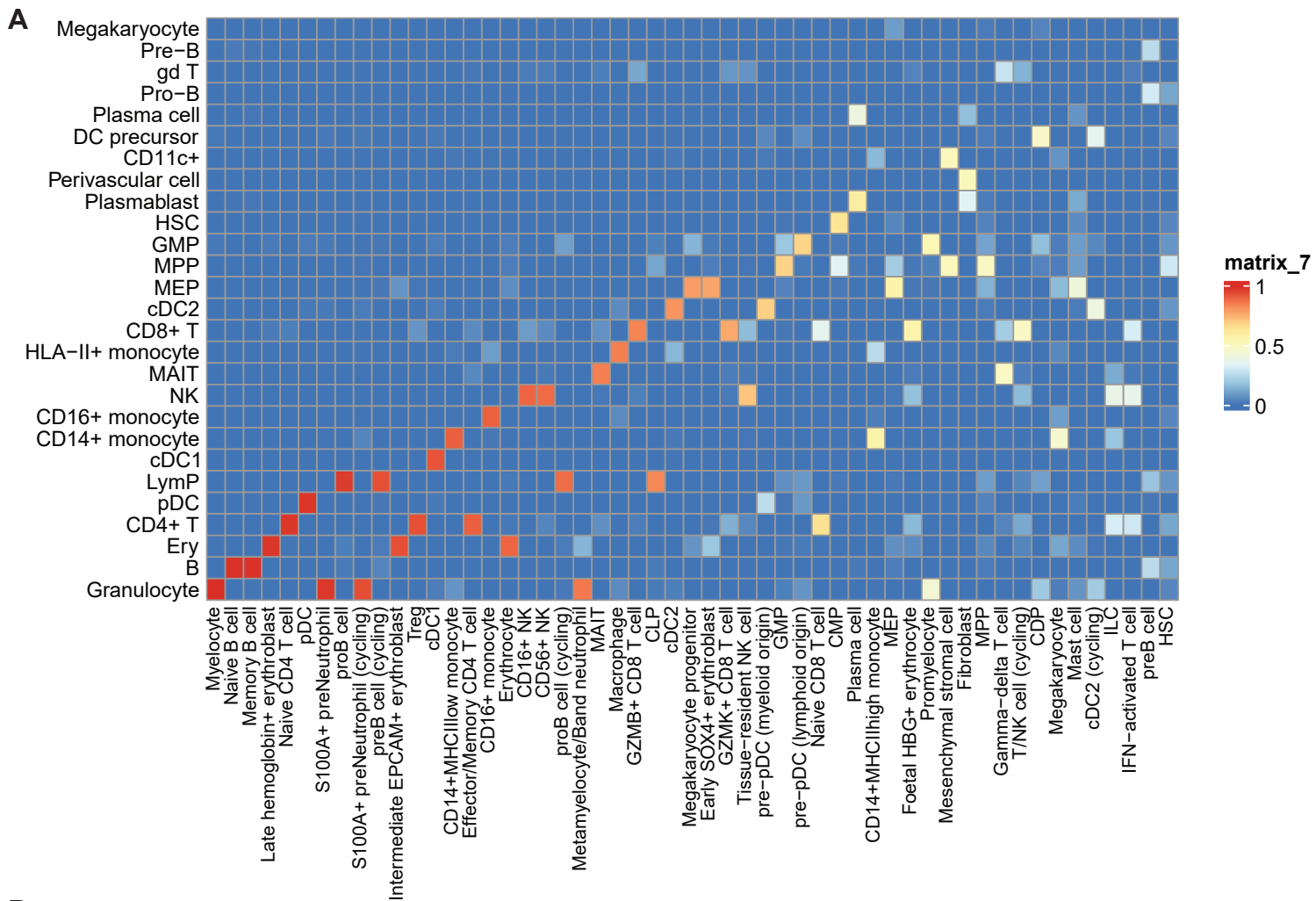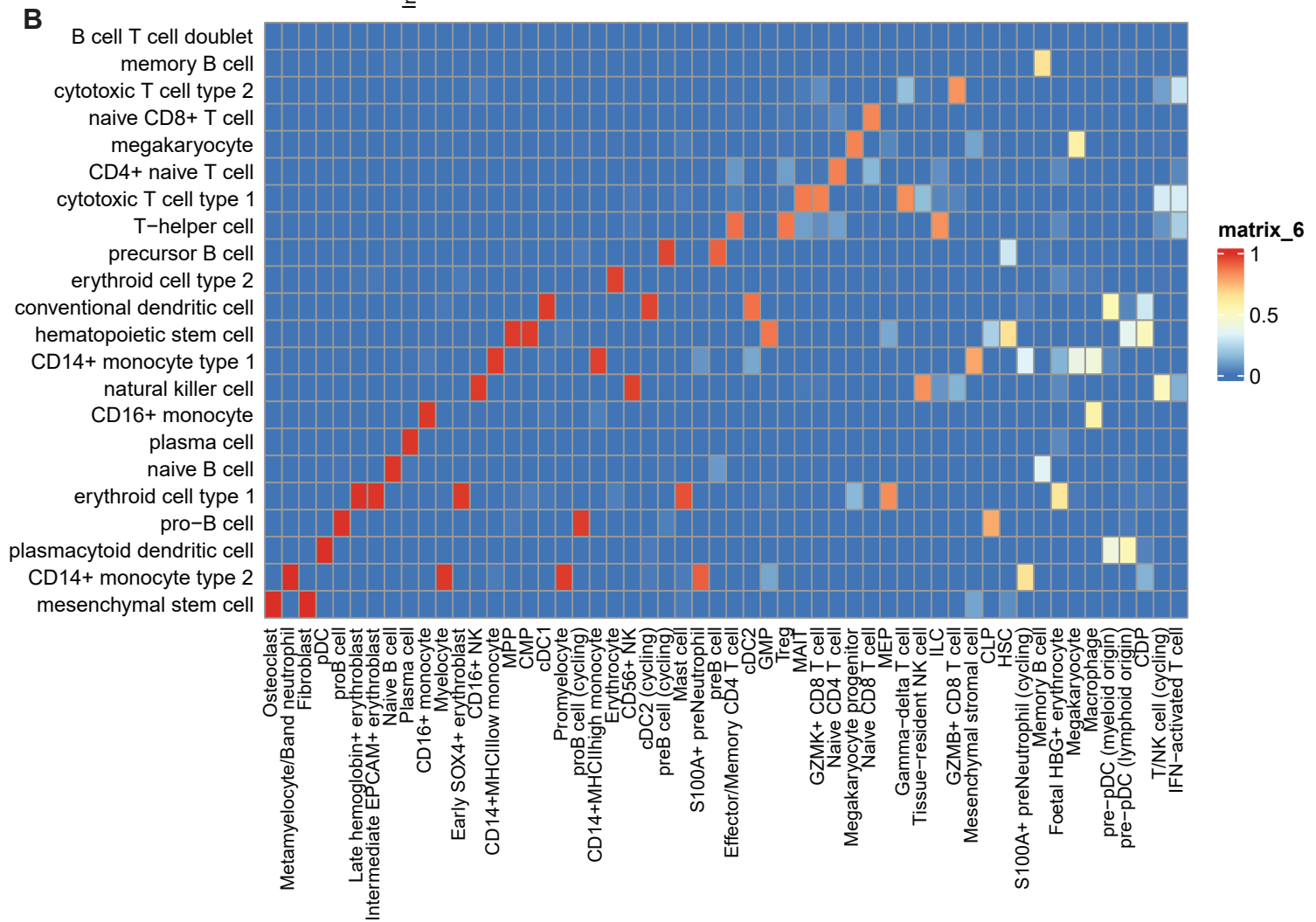

Supplement: Supplementary Figure 4 — Regression plot of naïve CD4+ T cell age correlation. Regression plots of cell type frequency changes with respect to age for all cell types in the bone marrow for naïve CD4+ T cells. [file DataSheet_4.pdf]

# Naive CD4 T cell

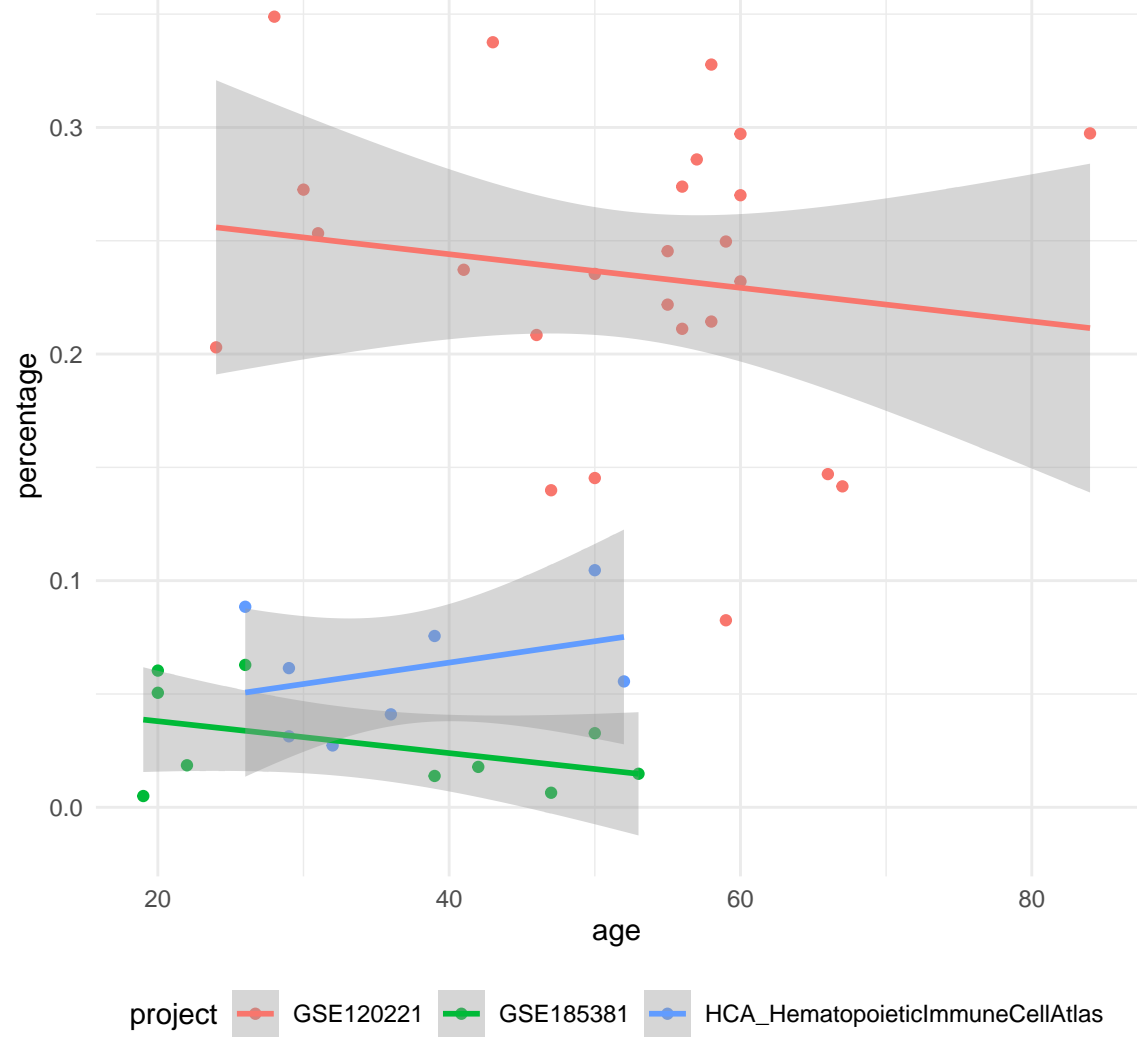

Supplement: Supplementary Figure 5 — Errors for 10-fold cross validation of cell age predictor. Median error of prediction computed in the 10-fold cross validation of cell age predictor. [file DataSheet_5.pdf]

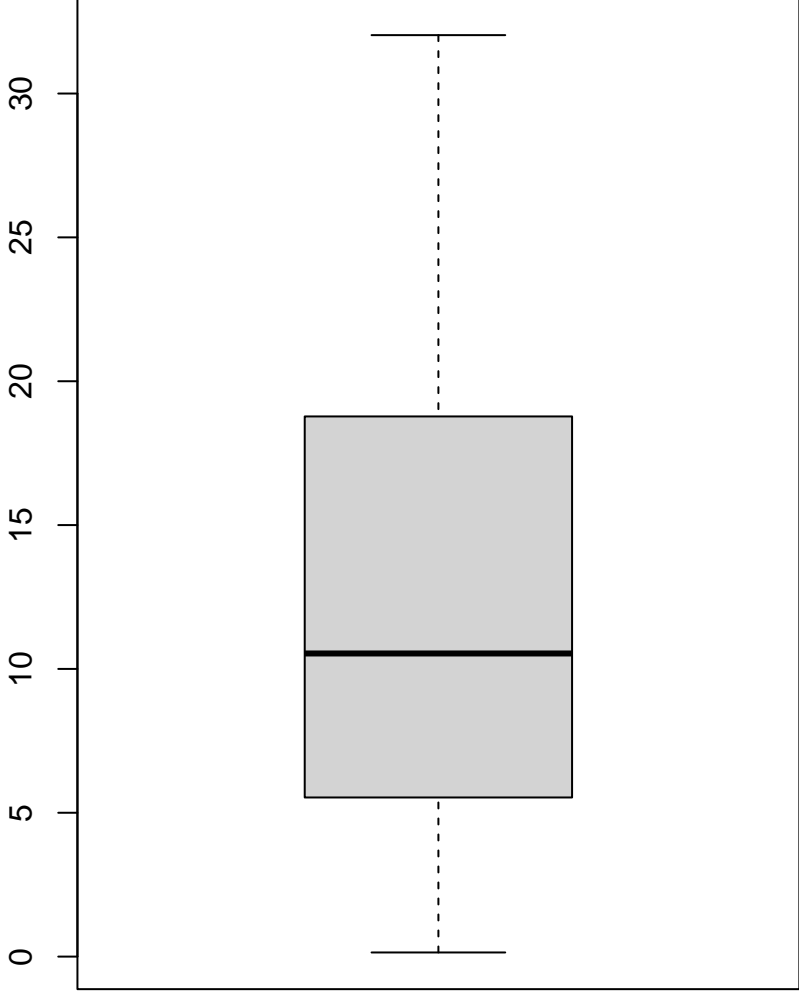

Supplement: Supplementary file 7 [file DataSheet_6.pdf]
