# Supplementary material for: Establishing a human bone marrow single cell reference atlas to study ageing and diseases
Source: Front Immunol. 2023 Mar 15;14:1127879. doi: 10.3389/fimmu.2023.1127879 (PMC10050687; doi:10.3389/fimmu.2023.1127879)
Supplement: Supplementary Figure 1 — (A) UMAPs of healthy BM samples before and after integration, coloured by project ID. (B) Comparison of cell type labels in the original studies (left) and our constructed bone marrow atlas (right). [file DataSheet_1.pdf]

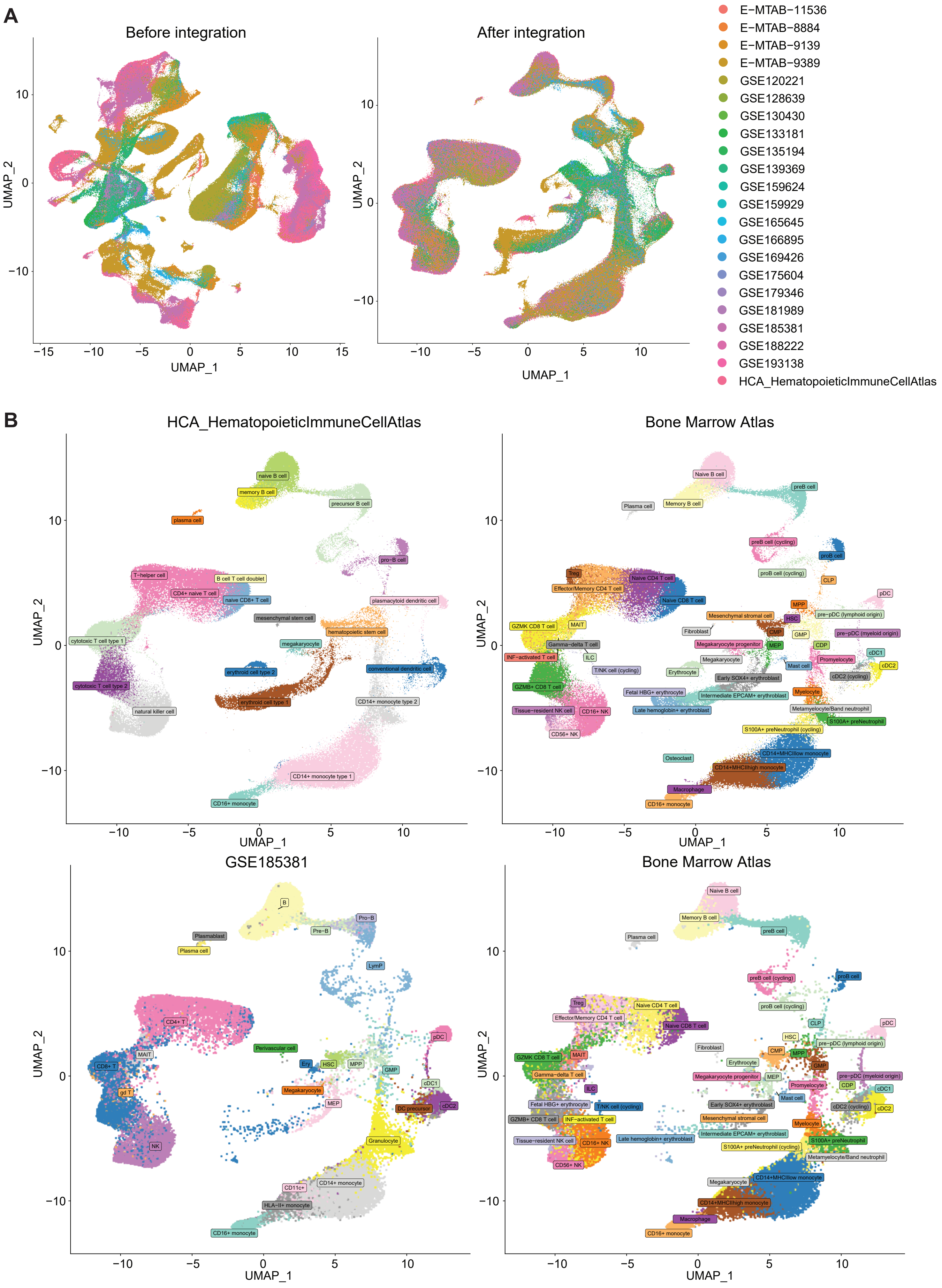
A
Before integration
After integration
UMAP_1
UMAP_2
E−MTAB−11536
E−MTAB−8884
E−MTAB−9139
E−MTAB−9389
GSE120221
GSE128639
GSE130430
GSE133181
GSE135194
GSE139369
GSE159624
GSE159929
GSE165645
GSE166895
GSE169426
GSE175604
GSE179346
GSE181989
GSE185381
GSE188222
GSE193138
HCA_HematopoieticImmuneCellAtlas
B
HCA_HematopoieticImmuneCellAtlas
naive B cell
memory B cell
precursor B cell
plasma cell
pro−B cell
T−helper cell
B cell T cell doublet
CD4+ naive T cell
naive CD8+ T cell
plasmacytoid dendritic cell
mesenchymal stem cell
hematopoietic stem cell
megakaryocyte
cytotoxic T cell type 1
erythroid cell type 2
conventional dendritic cell
cytotoxic T cell type 2
erythroid cell type 1
CD14+ monocyte type 2
natural killer cell
CD14+ monocyte type 1
CD16+ monocyte
Bone Marrow Atlas
Naive B cell
preB cell
Plasma cell
Memory B cell
preB cell (cycling)
proB cell
proB cell (cycling)
Treg
Naive CD4 T cell
CLP
Effector/Memory CD4 T cell
pDC
Naive CD8 T cell
MPP
Mesenchymal stromal cell
pre−pDC (lymphoid origin)
GZMK CD8 T cell
MAIT
HSC
Fibroblast
CMP
GMP
pre−pDC (myeloid origin)
Gamma−delta T cell
Megakaryocyte progenitor
MEP
CDP
INF−activated T cell
ILC
cDC1
Megakaryocyte
Promyelocyte
T/NK cell (cycling)
Mast cell
cDC2
Erythrocyte
cDC2 (cycling)
Early SOX4+ erythroblast
GZMB+ CD8 T cell
Fetal HBG+ erythrocyte
Myelocyte
Intermediate EPCAM+ erythroblast
Tissue−resident NK cell
CD16+ NK
Late hemoglobin+ erythroblast
Metamyelocyte/Band neutrophil
S100A+ preNeutrophil
CD56+ NK
S100A+ preNeutrophil (cycling)
Osteoclast
CD14+MHCIIlow monocyte
CD14+MHCIIhigh monocyte
Macrophage
CD16+ monocyte
GSE185381
B
Pro−B
Plasmablast
Pre−B
Plasma cell
LymP
CD4+ T
pDC
Perivascular cell
MAIT
CD8+ T
Ery
HSC
MPP
GMP
cDC1
gd T
Megakaryocyte
MEP
DC precursor
cDC2
Granulocyte
NK
CD11c+
CD14+ monocyte
HLA−II+ monocyte
CD16+ monocyte
Bone Marrow Atlas
