# Supplementary material for: Establishing a human bone marrow single cell reference atlas to study ageing and diseases
Source: Front Immunol. 2023 Mar 15;14:1127879. doi: 10.3389/fimmu.2023.1127879 (PMC10050687; doi:10.3389/fimmu.2023.1127879)
Supplement: Supplementary Figure 3 — Cell type annotation comparison. Heatmap comparing cell type annotation of constructed atlas and annotation from original samples. [file DataSheet_3.pdf]

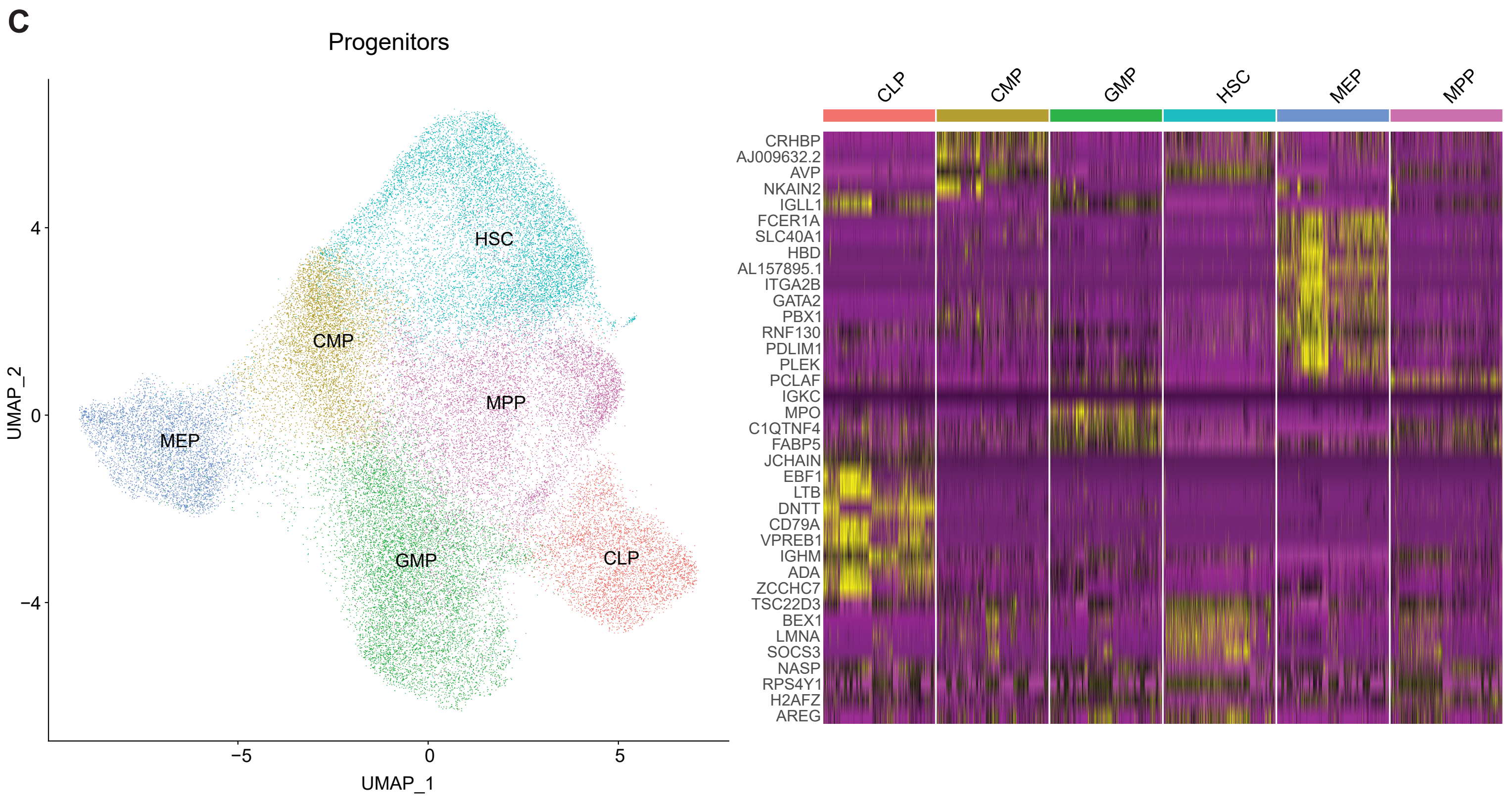
C
Progenitors
HSC
CMP
MPP
MEP
GMP
CLP
UMAP_2
UMAP_1
4
0
−4
−5
0
5
CLP
CMP
GMP
HSC
MEP
MPP
CRHBP
AJ009632.2
AVP
NKAIN2
IGLL1
FCER1A
SLC40A1
HBD
AL157895.1
ITGA2B
GATA2
PBX1
RNF130
PDLIM1
PLEK
PCLAF
IGKC
MPO
C1QTNF4
FABP5
JCHAIN
EBF1
LTB
DNTT
CD79A
VPREB1
IGHM
ADA
ZCCHC7
TSC22D3
BEX1
LMNA
SOCS3
NASP
RPS4Y1
H2AFZ
AREG

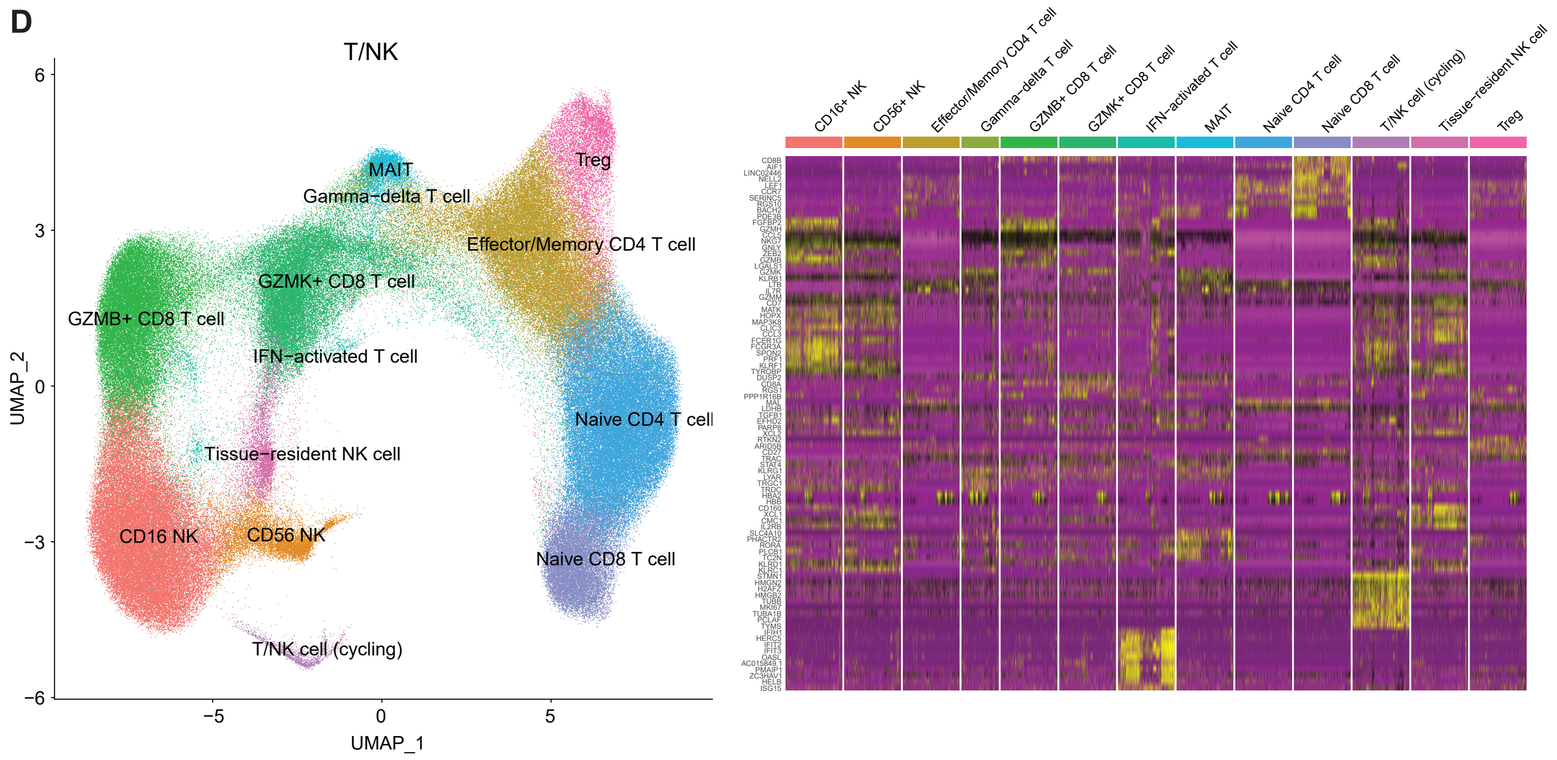
D
T/NK
MAIT
Treg
Gamma−delta T cell
Effector/Memory CD4 T cell
GZMK+ CD8 T cell
GZMB+ CD8 T cell
IFN−activated T cell
Naive CD4 T cell
Tissue−resident NK cell
CD16 NK
CD56 NK
Naive CD8 T cell
T/NK cell (cycling)
UMAP_2
UMAP_1
6
3
0
−3
−6
−5
0
5
CD16+ NK
CD56+ NK
Effector/Memory CD4 T cell
Gamma−delta T cell
GZMB+ CD8 T cell
GZMK+ CD8 T cell
IFN−activated T cell
MAIT
Naive CD4 T cell
Naive CD8 T cell
T/NK cell (cycling)
Tissue−resident NK cell
Treg
